# Peer Review of “The Impact of SARS-CoV-2 Lineages (Variants) and COVID-19 Vaccination on the COVID-19 Epidemic in South Africa: Regression Study”

**DOI:** 10.2196/46906

**Published:** 2023-07-03

**Authors:** Ramesh Poluru

**Affiliations:** 1 The INCLEN Trust International New Delhi India

**Keywords:** COVID-19, infection, pandemic, vaccine, vaccination, epidemiology, transmissibility, health care, hospital admission, COVID-19 variants, SARS-CoV-2

*This is a peer-review report submitted for the paper “The Impact of SARS-CoV-2 Lineages (Variants) and COVID-19 Vaccination on the COVID-19 Epidemic in South Africa: Regression Study.”*

## Round 1 Review

The manuscript [1] attempts to investigate the impact of SARS-CoV-2 lineages in South African COVID-19 epidemiology. I would like to congratulate the authors on this useful attempt. The manuscript is well written, and the subject addressed in this manuscript is worth investigating; however, the manuscript partly failed to present a clear picture of its analytical methodology and presentation of results. The following are some minor concerns for consideration. I suggest that the authors (a) extend the study to include the recent Omicron variant, (b) present results with complete models, (c) avoid excessive references (~71).

In conclusion, the subject addressed in this manuscript is worth investigating and acceptable after taking into account the abovementioned minor issues.
